# On-Field Evaluation of Mouthpiece-and-Helmet-Mounted Sensor Data from Head Kinematics in Football

**DOI:** 10.1007/s10439-024-03583-0

**Published:** 2024-07-25

**Authors:** Ty D. Holcomb, Madison E. Marks, N. Stewart Pritchard, Logan E. Miller, Steve Rowson, Garrett S. Bullock, Jillian E. Urban, Joel D. Stitzel

**Affiliations:** 1 Department of Biomedical Engineering, Wake Forest School of Medicine, 575 Patterson Avenue, Suite 530, Winston-Salem, NC 27101 USA; 2 Virginia Tech-Wake Forest School of Biomedical Engineering and Sciences, Winston-Salem, NC USA; 3 Department of Orthopedic Surgery and Rehabilitation, Wake Forest School of Medicine, Winston-Salem, NC USA

**Keywords:** Head impact exposure, Head kinematics, American Football, Instrumented mouthguard, Head Impact Telemetry System, Video review

## Abstract

**Purpose:**

Wearable sensors are used to measure head impact exposure in sports. The Head Impact Telemetry (HIT) System is a helmet-mounted system that has been commonly utilized to measure head impacts in American football. Advancements in sensor technology have fueled the development of alternative sensor methods such as instrumented mouthguards. The objective of this study was to compare peak magnitude measured from high school football athletes dually instrumented with the HIT System and a mouthpiece-based sensor system.

**Methods:**

Data was collected at all contact practices and competitions over a single season of spring football. Recorded events were observed and identified on video and paired using event timestamps. Paired events were further stratified by removing mouthpiece events with peak resultant linear acceleration below 10 g and events with contact to the facemask or body of athletes.

**Results:**

A total of 133 paired events were analyzed in the results. There was a median difference (mouthpiece subtracted from HIT System) in peak resultant linear and rotational acceleration for concurrently measured events of 7.3 g and 189 rad/s^2^. Greater magnitude events resulted in larger kinematic differences between sensors and a Bland Altman analysis found a mean bias of 8.8 g and 104 rad/s^2^, respectively.

**Conclusion:**

If the mouthpiece-based sensor is considered close to truth, the results of this study are consistent with previous HIT System validation studies indicating low error on average but high scatter across individual events. Future researchers should be mindful of sensor limitations when comparing results collected using varying sensor technologies.

## Introduction

In the United States, adolescents aged 15–19 years old are among those most likely to be hospitalized for an impairment related to traumatic brain injury (TBI) [1–3]. These injuries most often occur during participation in contact sports, with football being the leading cause of injury in male, high contact sports [4–6]. While concussive events remain a serious concern, focus has shifted to also include subconcussive head impacts [7–15]. A subconcussive head impact is defined as an impact to an athlete that does not result in signs or symptoms of a concussion [16]. Subconcussive head impacts in contact and collision sports are of increasing concern, as a growing body of research suggests that cumulative subconcussive head impacts can have an adverse effect on brain health, including changes in cognition, impairment of behavior, and alterations to the white matter structure of the brain [14, 17–22]. With approximately five million adolescent athletes participating in the sport of football each year, there is a critical need to understand and minimize head impacts [23–25].

Prior work has demonstrated that concussions can occur as a result of direct (i.e., head impact) or indirect (i.e., inertial head motion resulting from a collision to the body) impact to the head that causes a sudden change in the acceleration of the brain [26]. Only a small percentage (less than 1%) of head acceleration events (HAEs) that occur on the football field are associated with high risk for concussion [27–29]. The remainder of head impacts are considered ‘subconcussive’ in nature; the kinematics of subconcussive HAEs are generally lower in magnitude but occur more frequently than concussive HAEs. Therefore, HAEs should be accurately measured to better estimate injury risk and subconcussive head impact exposure.

In the past, researchers have utilized sensor systems to measure head impact exposure in contact sports [30–37]. Most notably, the Head Impact Telemetry (HIT) System (Simbex, Lebanon, NH) was the primary sensor system utilized to measure head kinematics resulting from HAEs in practices and competitions for over a decade [30–32, 38, 39]. This helmet-mounted sensor system consists of six accelerometers with a single system located within the existing space between padding in the helmet. The sensors are spring mounted, designed to remain in contact with the head, designed to compress with padding, and measure head kinematics during an HAE. Past studies have discussed the accuracy and precision of the HIT System and have found the data collected by the sensors to be acceptable [39]. The HIT System was previously demonstrated to be a reliable tool for collecting large quantities of data from multiple athletes over a single season and has been utilized in numerous head impact exposure studies at multiple levels of play in football [11, 24, 25, 30, 40–45]. Data collected by the HIT System has also resulted in the development of concussion risk functions used to estimate the likelihood of a concussion based on the peak resultant accelerations of an HAE [27, 46–49].

Recent advancements in sensor technology and the desire to collect HAE data in non-helmeted sports have fueled the development of alternative sensor attachment methods such as the X-Patch skin mounted device, instrumented headbands, and instrumented mouthguards [32, 50–52]. Such devices have been used to measure head acceleration kinematics in soccer, hockey, rugby, artistic gymnastics, and taekwondo, among other sports [53–58]. These devices often utilize a lower acceleration trigger threshold than the HIT System and have been used to quantify subconcussive HAEs occurring frequently at lower magnitudes [36]. Instrumented mouthguards are closer to the head center of gravity and provide superior coupling than skin-mounted or headband sensors resulting in more accurate data collection [36, 59]. However, few studies have been conducted to explore differences between antecedent and emerging sensor systems when measuring HAEs concurrently in the same athlete [50, 60]. Previous work has also shown that a wearable sensor’s performance in-lab is not always indicative of on-field performance [50]. It is valuable to compare instrumentation approaches to ensure that the kinematics measured are in agreement and to gain a better understanding of how separate systems measure the same HAE. Therefore, the objective of this study was to compare peak resultant magnitude of head acceleration events measured from athletes dually instrumented with the HIT System and a mouthpiece sensor system.

## Materials and Methods

A total of five high school football athletes (ages 15–18) were enrolled in this study, approved by the Wake Forest University School of Medicine Institutional Review Board (IRB00049715). All minor study participants completed assent forms and written consent was obtained from a legal guardian. Written consent was obtained from all athletes over the age of 18. The athletes were fitted with either a Riddell Speed or Speed Flex football helmet containing a HIT System MxEncoder with 5 degrees-of-freedom and simultaneously instrumented with a custom-fit mouthpiece containing the mouthpiece sensor system with a bonded mouthguard [36]. The MxEncoder consisted of a digital encoder, accelerometers, and instrumentation to transmit signals to a base unit monitored by a member of the research team during a session [61, 62]. The mouthpiece sensor system utilizes an accelerometer and an angular rate sensor mounted in a retainer style enclosure custom fit to the athlete’s upper palate and dentition to measure six degree head kinematics [36]. Prior to the season, the palate and dentition of each athlete was collected via a digital scan (TRIOS intraoral scanners; 3Shape A/S, Copenhagen, Denmark). A high-resolution 3D print of the upper palate and dentition was used to create the custom mouthpiece for each athlete and ensure optimal coupling with the teeth. To comply with mouthguard regulations in football, a soft mouthguard overlay was molded and affixed to the custom acrylic mouthpiece. The athletes were instrumented for a single spring football season.

### Sensor Settings

The HIT System was set to record at the system default 10 g single axis trigger threshold while the mouthpiece sensors were set to record an event when 5 g was exceeded on any axis for at least 3 ms. The mouthpiece sensors recorded data 15 milliseconds prior, and 45 milliseconds post trigger. The sampling rate for the accelerometer was 4681 Hz and the sampling rate for the gyroscope was 860 Hz for the mouthpiece-based sensors. The sampling rate was 1000 Hz for the HIT System. The rotational scaling factor recommended by Rowson et al was applied to the rotational acceleration measured by the HIT System [46]. All mouthpiece data were transformed to the head center of gravity (CG). The HIT System data was translated to the head CG [63].

### On-Field Data Collection

Trained researchers monitored the HIT System and mouthpiece-based sensors at all contact practices and competitions. Two cameras were used to capture time-synchronized film at each session with a camera placed at the end zone and on the sideline for the best possible view of study participants. These cameras were placed and activated approximately 15–30 min prior to the session start and the video was time aligned to the mouthpiece client. To complete this time alignment, the tablet with the time used by the mouthpiece client was filmed prior to the beginning of the recording to ensure that the first frame captured by the video would be of the mouthpiece client time (HH:MM:SS). This first frame of the mouthpiece client time was used to pair the recorded events to the session video collected by the two cameras for video review at the conclusion of the season. Events measured by the HIT System were paired to mouthpiece sensor events based on independently coded contact characteristics. The mouthpiece and HIT System were time aligned using the time stamps of events associated with contact characteristics as described below.

### Video Analysis and Pairing Events

Using a custom video analysis program developed in MATLAB (MathWorks, Natick, MA), recorded events were observed and identified on video and paired semi-automatically with video time corresponding to event timestamp [64]. For the purpose of this study, all recorded events across both sensor systems were independently video verified using this process. Events not in the field of view of the video were excluded. If an event was excluded from the data set, the reason for exclusion was provided.

Events were independently reviewed and paired between devices using the measured event recording time and the synchronized video time. To control for potential time drift between sensor systems, video reviewers identified the exact time of two clearly visible and unobstructed events to act as anchor points. The frame of the video associated with each anchor point was recorded and used to time synchronize the remaining events with video assuming a linear time drift between the video and the sensor system. A time stamp was assigned to each remaining video-verified event at the exact moment of contact observed on video using the new time aligned offset generated by the two clearly visible events. Events from the HIT System and the mouthpiece were then paired if they were associated with the same frame of video. A tolerance window of 1 s (− 0.5 s, + 0.5 s) was allowed when pairing events by video frame to account for errors in the assumption of a linear time drift. Once any events were identified within the 1 s time window, the window was further reduced to ensure the event with the closest event recording time were paired as a matching event.

### Statistical Analysis

An HAE is defined as an event that causes an acceleration response of the head caused by an external short-duration collision force applied directly to the head or indirectly via the body in sport [65]. Wearable devices are often validated for direct HAEs and not indirect HAEs due to the limitation of reproducing indirect HAEs in the lab and identifying indirect HAEs on the field [65]. The HIT System was designed to measure head impacts where spring-loaded accelerometers located in the helmet and pushed into the head [30, 31, 66, 67]. During contact to the body, the spring-loaded accelerometers can decouple from the head. To make a fair comparison between sensor systems, only events where direct contact to the helmet of the athlete was observed on video were included in the paired events. Additionally, all events where the athlete experienced contact on the facemask of the helmet were excluded from the data set, as prior studies have shown larger error in HIT System measurements for this impact location [30, 31]. Paired events where the mouthpiece measured a peak resultant linear acceleration of less than 10 g were excluded. Peak resultant linear and rotational acceleration were calculated for each recorded event and compared across sensor systems. Data were quantified in terms of number of true-positives (i.e., video verified sensor-recorded event), mean peak resultant linear and rotational acceleration, median peak resultant linear and rotational acceleration, and 95th percentile peak linear and rotational acceleration for each device using JMP, Version 15.0.0 (SAS Institute Inc, Cary, NC, 1989–2022). Mixed effect models were used to evaluate differences in paired event kinematics between sensors (SAS Statistical Analysis Software Version 9.4).

The difference in peak resultant linear and rotational acceleration was calculated across sensor systems by subtracting the mouthpiece sensor event data from the HIT System sensor event data. Mean peak resultant linear and rotational acceleration, median peak resultant linear and rotational acceleration, and 95th percentile peak linear and rotational acceleration for each device was compared and histograms were used assess differences in sensor measurements. A Bland Altman analysis for agreement was performed to assess the measured kinematics offset between sensor systems for all paired events [68].

## Results

A total of 6835 events were collected with the mouthpiece sensor system and a total of 1591 events were collected with the HIT System over the course of 33 sessions. Events were collected from athletes in a variety of positions on offense and defense including a running back, a wide receiver, a defensive lineman, and two linebackers. Using video verification, 5,668 false-positive events (82.9%) were excluded from the mouthpiece sensor data and 768 (48.3%) false-positive events were excluded from the HIT System data. A false-positive event was often the result of an athlete or research staff handling the sensor off-field (e.g., removing a helmet or a mouthpiece while on the sidelines or during a water break). In total, 1167 true-positive head acceleration events (17.1%) were identified from the mouthpiece sensor data and 823 true-positive head acceleration events (51.7%) were observed from the HIT System data. These events were then paired based on the time of the recorded event coincident with observed contact in video; 471 events were captured by both sensor systems.

**Fig. 1:**
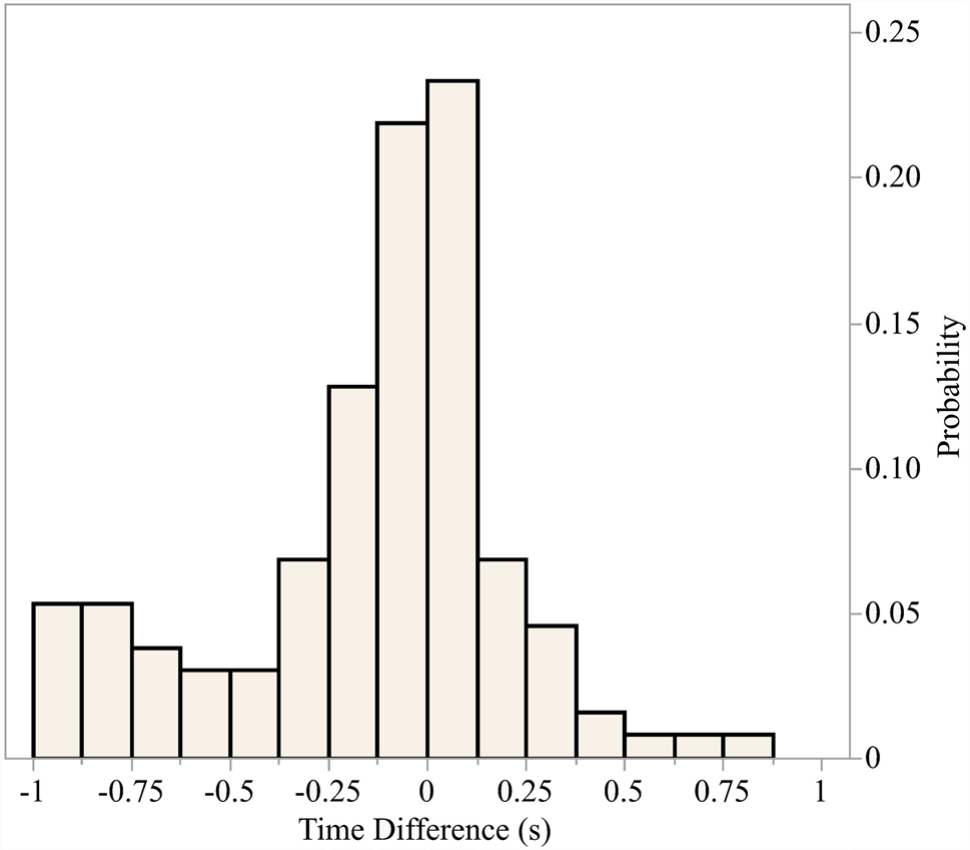
Time stamping distribution for paired events (*n* = 471)

The 471 paired events were evaluated and paired using time stamps placed at the exact moment of contact using session film. A histogram of the distribution of time between paired event stamps is presented in Fig. 1. Approximately 70% of the time stamp differences fell between ± 0.30 s. Of the 471 paired events, 39 events (8.3%) had the potential to be paired to multiple events within the 1 s time window. The remaining 432 events (91.7%) had only a single event with which to paired on the opposing sensor system.

**Fig. 2:**
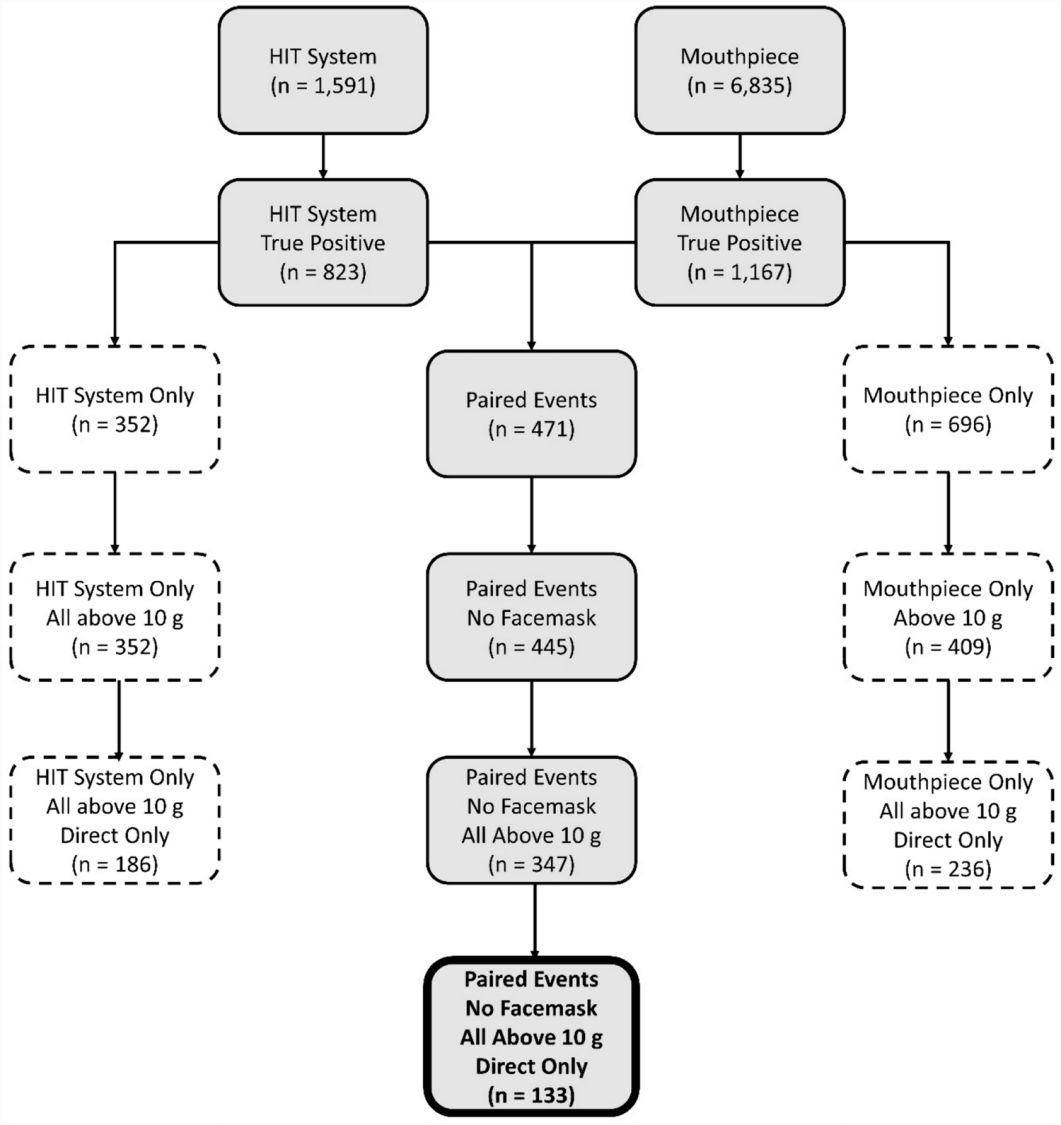
Flow chart of total collected events and included number of events

Of the 471 paired events measured by both sensor systems, 26 paired events (5.5%) were excluded due to video-verified direct contact with the facemask of the helmeted athlete. 98 of the remaining 445 paired events (22.0%) were excluded as the mouthpiece measured a peak resultant linear acceleration lower than 10 g. An additional 214 of the remaining 347 paired events (61.7%) were excluded as they were a result of inertial head motion due to contact with the body of the athlete and not direct contact to the head. The data set presented in the following tables and figures are in reference to the remaining 133 paired events. Figure 2 provides a flow chart of data reduction for analysis.

A total of 352 events (22.1%) were recorded by the HIT System sensors and not the mouthpiece. Because of the HIT System trigger threshold, all events recorded by the HIT System were greater than 10 g. These 352 HIT System-only events had an average peak resultant linear acceleration of 24.6 g with a median of 18.0 g and a 95th percentile of 56.4 g. The average peak resultant rotational acceleration was 1108 rad/s^2^ with a median of 863 rad/s^2^ and a 95th percentile of 2755 rad/s^2^. Additionally, a total of 696 events (9.4%) were recorded by the mouthpiece sensors and not the HIT System. Of these 696 events, 409 events (58.7%) were above 10 g. These 409 mouthpiece-only events over the 10 g threshold had an average peak resultant linear acceleration of 20.0 g with a median of 15.5 g and a 95th percentile of 43.2 g. The average peak resultant rotational acceleration was 1116 rad/s^2^ with a median of 833 rad/s^2^ and a 95th percentile of 2458 rad/s^2^. A total of 133 events were measured concurrently by both sensor systems and categorized as direct head events with no facemask contact, and above 10 g. Linear regressions for the peak resultant linear and rotational accelerations are provided in Appendix 1, Fig. 6A. Table 1 includes the summary statistics for these paired events. Difference (HITS-MP) was calculated on a per event basis. The summary statistics represent the distribution of differences.

**Table 1:** Summary statistics of peak resultant linear and rotational acceleration for paired events (*n* = 133)

Kinematic	Metric	Mean	95% CI	SD	Median	95 percentile
Linear acceleration (g)	Mouthpiece	22.1	19.8, 24.5	13.7	17.9	50.6
	HITS	31.0	27.8, 34.2	18.6	25.7	65.5
	Diff. (HITS-MP)	8.8	5.4, 12.3	20.2	7.3	40.0
Rotational acceleration (rad/s^2^)	Mouthpiece	1226	1071, 1381	903	990	2764
	HITS	1329	1170, 1489	930	1074	3378
	Diff. (HITS-MP)	104	− 103, 311	1207	189	2031

**Fig. 3:**
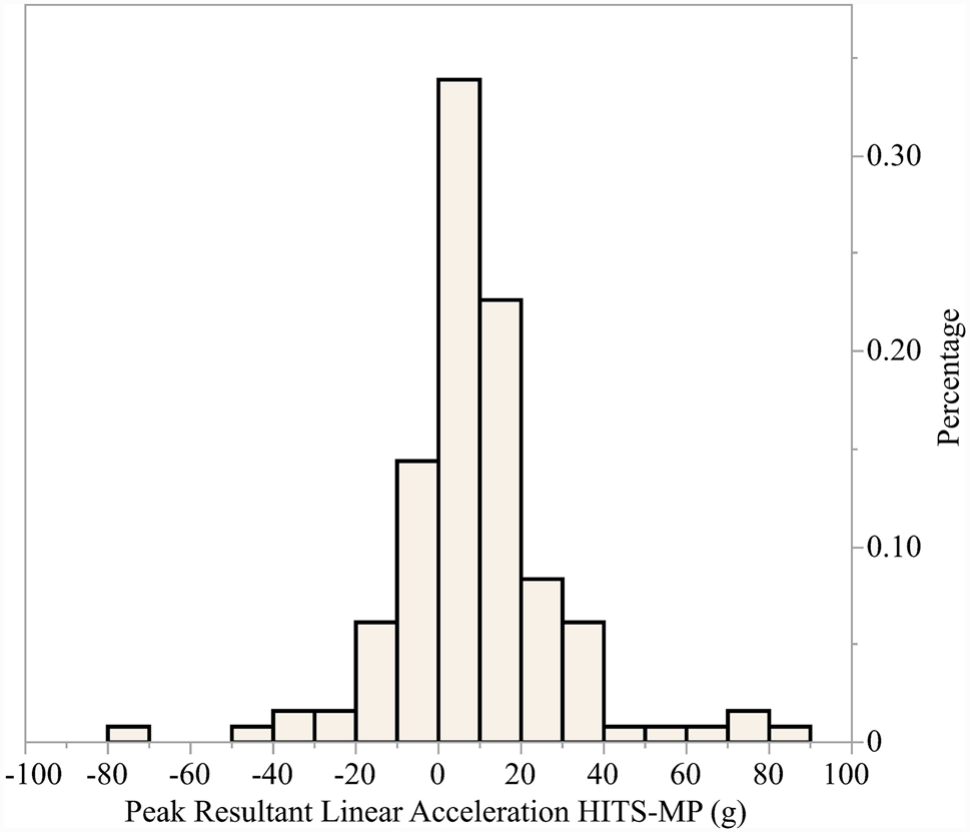
Paired event sensor system comparison (*n* = 133) Histogram of difference in peak resultant linear acceleration (HITS minus MP)

The peak resultant linear acceleration and rotational acceleration recorded by each sensor system for the 133 paired events are presented in Fig. 3. Figure 3 is a histogram of the magnitude difference in peak resultant linear acceleration between the sensor systems (HIT System data subtracted from the mouthpiece sensor data); approximately 90% of the differences fell between ± 40 g. The mean peak resultant linear acceleration of the HIT System was significantly greater than that of the mouthpiece sensors (*p* = 0.006).

**Fig. 4:**
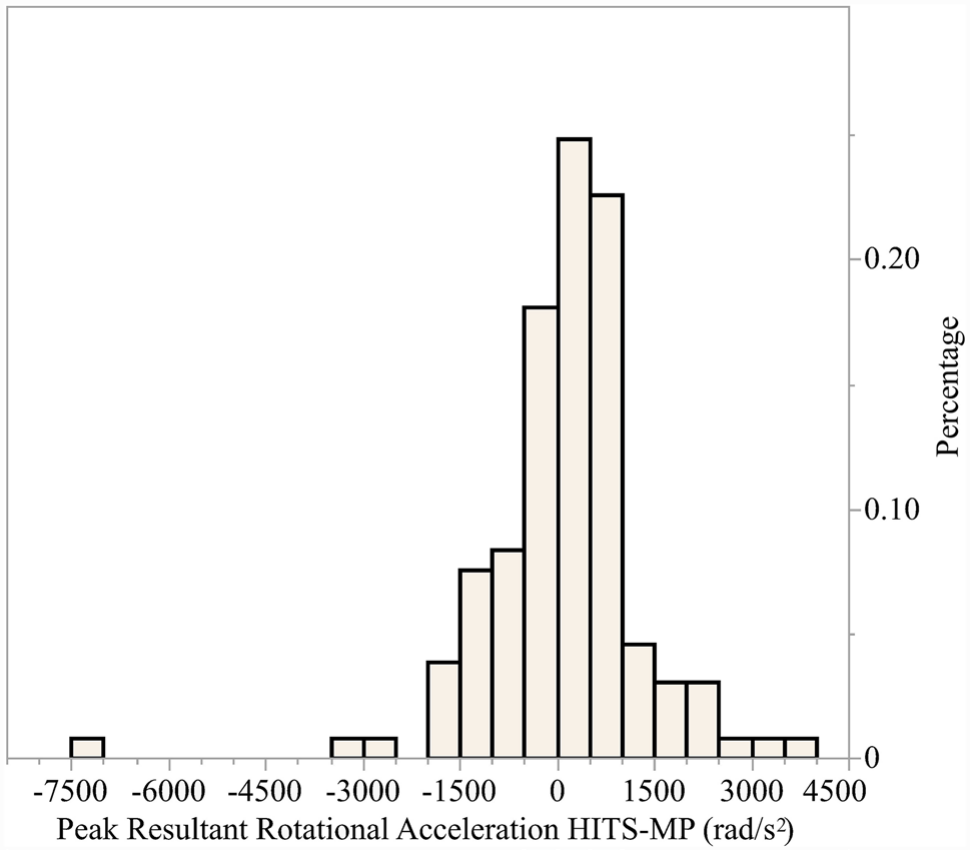
Paired event sensor system comparison (*n* = 133) Histogram of difference in peak resultant rotational acceleration (HITS minus MP)

The peak resultant rotational acceleration recorded by each sensor system for the 133 paired events is presented in Fig. 4. The mean peak resultant rotational acceleration of the HIT System was not significantly greater than the mean of the mouthpiece sensor (*p* = 0.96). Figure 4 provides a histogram of the difference in peak resultant rotational acceleration between the sensor systems and approximately 90% of the paired events fell between ± 1500 rad/s^2^ difference in peak resultant rotational acceleration.

### Bland–Altman Analysis

**Fig. 5:**
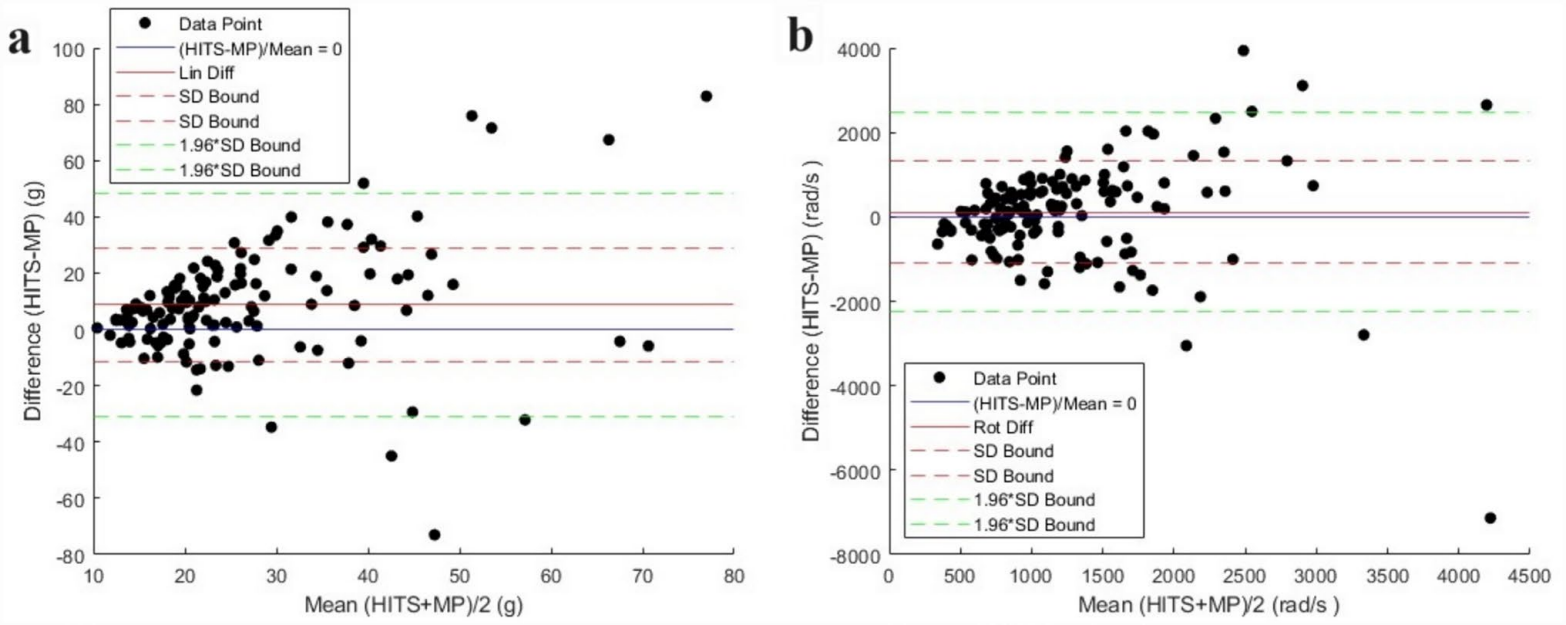
Bland–Altman analysis for agreement plots (*n* = 133). **a** Peak resultant linear acceleration. **b** Peak resultant rotational acceleration

**Fig. 6:**
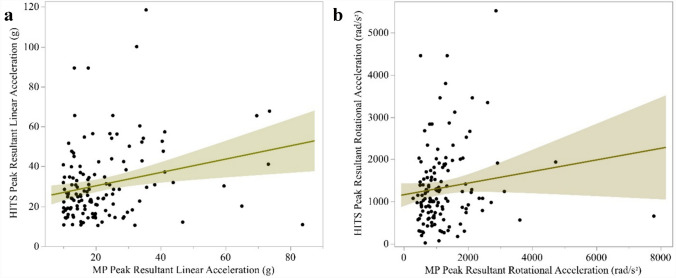
Scatterplot of paired events sensor system comparison (*n* = 133). **A** Peak resultant linear acceleration. **B** Peak resultant rotational acceleration

A Bland–Altman analysis for agreement was performed on the 133 paired events. The x-axis on each plot is the average between the HIT System and mouthpiece measurements. The y-axis on each plot is the difference in engineering units between the two systems with the mouthpiece measurement subtracted from the HIT System measurement. The solid red line represents the mean bias, and the dashed red lines represent the bounds of the limits of agreement of the standard deviation of the differences between sensor systems. The dashed green line represents the upper and lower limits of agreement, defined as the range of the difference in which 95% of measurements are expected to fall within. Figure 5a is a Bland–Altman plot of the peak resultant linear acceleration. A mean bias ± SD between the paired events measured by HIT System and the mouthpiece-based sensors of 8.8 g ± 20.2 was observed and the limits of agreement (LoA) (lower CI, upper CI) were − 30.7 (− 35.7, − 25.8) and 48.4 (43.4, 53.4) for peak resultant linear acceleration with the HIT System measuring greater linear acceleration on average for the paired event. Figure 5b is a Bland–Altman plot of the peak resultant rotational acceleration. The regression beta (lower CI, upper CI) was 0.48 (0.22, 0.74) for peak resultant linear acceleration. A mean bias ± SD of 104 rad/s^2^ ± 1207 was noted between sensor systems and the LoA (lower CI, upper CI) were − 2262 (− 2559, − 1965) and 2469 (2172, 2766) with the HIT System having the tendency to measure the greater value for peak resultant rotational acceleration. The regression beta (lower CI, upper CI) was 0.05 (0.02, 0.07) for peak resultant rotational acceleration.

## Discussion

The objective of this study was to compare peak resultant magnitude of head acceleration events measured from athletes dually instrumented with the HIT System and a mouthpiece sensor system. After excluding events where the mouthpiece measured peak resultant linear acceleration lower than 10 g and events where the facemask of the athlete was contacted, the mouthpiece sensors recorded lower peak linear and rotational acceleration magnitudes, measuring a median peak resultant linear acceleration of 17.9 g and a median rotational acceleration of 990 rad/s^2^ compared to a median peak resultant linear and rotational acceleration of 25.7 g and 1074 rad/s^2^ measured by the HIT System sensors.

The HIT System has been utilized in previous studies to evaluate head impact exposure in football. Prior studies collected events from high school football team athletes using the HIT System have reported consistent results [42, 69]. The mean peak linear acceleration and peak rotational acceleration reported for athletes from previous HIT System studies were similar to the results measured by the HIT System sensors in this study. Mean linear acceleration ranged from 21.7 to 28.6 g. Mean peak rotational acceleration ranged from 1232 to 1789 rad/s^2^ [42, 69]. The mean kinematic values measured by the HIT System in this study are comparable to these previous results with the HIT System measuring a mean peak resultant linear acceleration of 31.0 g and a mean peak resultant rotational acceleration of 1329 rad/s^2^. Two studies have been conducted to evaluate head impact exposure in youth football using the same mouthpiece sensors used in this study [70, 71]. One study reported a median peak resultant linear acceleration 14.7 g and a median peak resultant rotational acceleration of 954.6 rad/s^2^ for all events greater than 10 g [70]. The results of this study are comparable to the previous mouthpiece sensor studies; however, the differences in summary statistics are likely due to differences between levels of play and a lower sample size of athletes.

A median difference in magnitude between sensor systems (mouthpiece subtracted from HIT System) of 7.3 g for peak resultant linear acceleration and a median difference in magnitude of 189 rad/s^2^ for peak resultant rotational acceleration was recorded. The mean difference is relatively low and falls within ranges provided in previous studies examining head impact exposure in football [42, 69–71]. A greater difference in event magnitude was observed when high magnitude events were measured by at least one of the sensor systems. A Bland–Altman analysis for agreement was used to assess variance and scatter within the data set. The Bland–Altman analysis highlights the tendency for the different sensors to measure similar values for lower magnitude events below 20 g. As the magnitude of the events increase, the scatter of the data increases as well with a mean bias of 8.8 g and 104 rad/s^2^ and beta coefficient of 0.48 and 0.05, respectively. The results demonstrate a systematic bias with the mouthpiece-based sensor more likely to measure a lower magnitude than the HIT System for both peak resultant linear acceleration (LoA of − 30.7 and 48.4) and peak resultant rotational acceleration (LoA of − 2262 and 2469). Because data in this study was collected in the field, we cannot determine if one sensor system is more accurate in measuring the forces experienced at the head center of gravity, however, we can conclude that systematic bias exists between systems for events measured concurrently by both sensor systems.

A total of 352 events were captured by the HIT System and not the mouthpiece-based sensors and a total of 696 events were captured by the mouthpiece-based sensors and not the HIT System. It is possible that a 10 g single axis trigger could miss an event up to approximately 17 g peak resultant linear acceleration if all three axes (*x*, *y*, and *z*) measured approximately 9.8 g. While this is an edge case, it could explain other instances where the HIT System sensors failed to capture an event that the mouthpiece-based sensor captured because of the difference in trigger threshold. The lower 5 g threshold used by the mouthpiece resulted in a greater total of collected head acceleration events when resultant peak linear acceleration was greater than at least 8.5 g. Additionally, after a sensor system is triggered and records a potential event, the device must progress through a refractory period before being ready to collect data again. Differences in refractory period between the mouthpiece-based sensors and the HIT System may also explain events that were captured by only one sensor system.

A similar comparison study was conducted by Shah et al, dually instrumenting two athletes with both the intelligent mouthguard (IMG; Prevent Biomechanics, Edina, MN) and the HIT System over the course of a single football session with both devices set to record events that surpassed a 10 g threshold [60]. The results of the IMG/HIT System study were similar to the results of this study, with the HIT System generally measuring greater values for peak resultant linear acceleration. However, the IMG measured greater values for peak resultant rotational acceleration. This may be due to the difference in sampling rate between the mouthguard and the HIT System. The HIT System collected twice the number of events as the IMG and the authors speculated that the IMG did not record as many events as the HIT System due to video-verified contact not meeting the required 10 g threshold. The results of this study corroborate this speculation, as 471 events captured by the mouthpiece sensors were paired with events recorded by the HIT System sensors before removing paired events lower than 10 g as shown in Fig. 1. A total of 98 paired events were excluded from the analysis because the mouthpiece event was lower than 10 g. However, the mouthpiece recorded 696 events not recorded by the HIT System sensor, of which 287 events (41.2%) were below 10 g, while the HIT System only recorded 352 true-positive video-verified events not recorded by the mouthpiece. This difference in total events not recorded by the opposing sensor could be due in part to the different coupling methods utilized by both sensor systems [59, 72]. The mouthpiece is coupled to the upper palate and dentition of the athlete [36, 70], while the HIT System is located in the helmet and relies on a good fit to the head of the athlete [30, 31, 72].

Prior work evaluating the HIT System have identified that the sensor is a good instrument for performing analyses on large distributions of data collected from multiple athletes on a team or over the course of several sport seasons [30, 42, 45, 73, 74]. The results of this study are consistent with these previous findings. Individual head impact events are more difficult to accurately measure with the HIT System as the location of the sensor between the padding of the helmet can cause the sensor to overpredict the severity of an event [31, 59, 72, 75]. Instrumented mouthguards have been compared to helmet-based sensors in a lab setting using a NOCSAE headform [36, 59]. In a validation study conducted by Rich et al, the mouthpiece-based sensor showed good agreement (*R*^2^ = 0.95 for linear acceleration and *R*^2^ = 0.97 for rotational acceleration) for individual helmeted and un-helmeted events. An additional study compared a mouthpiece-based accelerometer to a helmet-mounted accelerometer in a lab testing environment and found that the mouthpiece accelerometer was more closely related and more accurate in measuring the forces experienced by the head center of gravity than the helmet-mounted sensor [59]. The differences between in-lab testing results and the on-field results from this study may be a result of loose chinstraps or poor helmet fitting. Additionally, the mouthpiece sensors have previously measured high frequency events in the field that can overpredict peak resultant rotational acceleration and peak resultant linear acceleration [70, 71]. Current recommendations state that all head kinematic data should be filtered to remove spurious frequencies to maximize the removal of noise while preserving impact signal [51]. Recent consensus on the best practices for collecting wearable sensor data recommends use of video verification in tandem with sensor data to authenticate events measured by the sensor system [51, 65]. The HIT System remains a strong scientific tool for measuring and evaluating large datasets while the mouthpiece-based sensor is stronger for evaluation of individual mouthpiece events. Researchers should be mindful of sensor limitations and potential for measurement errors when comparing results collected using varying sensor systems and methodologies and should strive to use best practices such as video-verification when collecting HAEs in an on-field environment.

The study includes several limitations. Only five high school varsity athletes were dually instrumented with both sensor systems for the purpose of this study. The mouthpiece sensor and HIT System had different trigger thresholds with the mouthpiece recording an event when any accelerometer axis exceeded 5 g while the HIT System recorded an event when the accelerometers between the helmet padding exceeded 10 g. The exclusion of paired events where the peak resultant linear acceleration measured by the mouthpiece was lower than 10 aimed to account for this limitation, but future studies could utilize the same threshold for both sensor systems insofar as this can be achieved. A time drift was identified between sensor systems. Steps were taken to mitigate the effect of time drift, including the use of linear offset video-to-sensor time alignment using clearly visible and unobstructed events in tandem with time stamping video-verified events. Approximately 70% of paired events had a difference between time stamps of 0.30 s. A time window of 1 s (− 0.5, + 0.5) was used to ensure events were paired with accuracy. However, future studies could improve this methodology and produce a more effective method to synchronize sensor systems. Data collected from both systems was filtered and differences in filtering methods could account for differences between paired events. Events were verified as true-positive using video-verification by a single reviewer which introduces potential for human bias. Data collected in this study was from a single high school varsity football team and thus may not be generalizable to all football teams across the country.

## Conclusions

Previous studies have evaluated helmet sensors and mouthpiece sensors using in-lab testing and concluded that helmet fit, and skull coupling could result in disparities between sensor systems. The results of this study corroborate these findings, demonstrating a difference in the HIT System and mouthpiece sensors. The data shows that while the mean difference between peak resultant linear acceleration measured by each device is statistically significant. Because data in this study was collected in the field, we cannot determine if one sensor system is more accurate in measuring the forces experienced at the head center of gravity but can conclude that systematic bias exists between the systems. However, since the majority of HAEs collected in football are lower in magnitude, these differences are minimized when evaluated on aggregate for larger quantities of data collected with either sensor over the course of a football season. The HIT System continues to be a strong scientific tool for analyses on large datasets while mouthpiece-based sensors are stronger for evaluation of individual mouthpiece events which is consistent with previous laboratory findings. Researchers should be mindful of sensor limitations and potential for measurement errors when comparing results collected using varying available sensor technologies. Video-verification of HAEs in tandem data collected from wearable sensors can increase confidence in exposure statistics. The results of this study may inform interpretation of head acceleration data collected from various sensors and aid in the interpretation of past study results that utilized preceding sensor systems.

## Appendix 1

See Fig. 6.
